# T-bet: biological functions, molecular mechanisms, and therapeutic applications: a systematic review

**DOI:** 10.3389/fimmu.2026.1671806

**Published:** 2026-01-23

**Authors:** Xiaowen Yang, Min Sun, Xinyi Tang, Xiaoyuan Zhang, Wenzhi Shen

**Affiliations:** 1 Shandong Provincial Precision Medicine Laboratory for Chronic Non-communicable Diseases, Institute of Precision Medicine, Jining Medical University, Jining, China; 2 College of Integrative Chinese and Western Medicine, Jining Medical University, Jining, Shandong, China

**Keywords:** cancer, immunotherapy, non-neoplastic diseases, single nucleotide polymorphism (SNP), T-bet

## Abstract

T-bet is a transcription factor predominantly expressed in immune cells, and it has been associated with a range of physiological and pathological processes, including the differentiation of various immune cell types, the development of immune-related diseases, and tumor progression. Despite notable advancements in the field, current research on T-bet remains fragmented, primarily concentrating on functional studies within specific cell types or the progression of particular diseases. This review aims to provide a comprehensive synthesis of the most recent findings regarding the role of T-bet in various diseases, with an emphasis on elucidating its molecular mechanisms and potential clinical applications. We underscore the involvement of T-bet in the pathogenesis of systemic diseases, including autoimmune disorders, infectious diseases, allergic conditions, endocrine disorders, psychiatric illnesses, and chromosomal abnormalities. Furthermore, we summarize its role in the development of various malignant tumors, such as esophageal cancer, gastric cancer, breast cancer, colon cancer, prostate cancer, and hematological malignancies. Additionally, we discuss the impact of T-bet on several critical processes in tumor biology, including tumor cell proliferation, apoptosis, epithelial-mesenchymal transition (EMT), metastasis, immune cell infiltration, and iron-induced apoptosis. We also assess the potential of T-bet as a prognostic and therapeutic target for tumors. In conclusion, T-bet may serve as a significant biomarker for the diagnosis and treatment of immune disorders and cancer, as well as a target for innovative immunotherapeutic strategies aimed at addressing tumors and immune-related diseases.

## 1 Introduction

**Figure 1:**
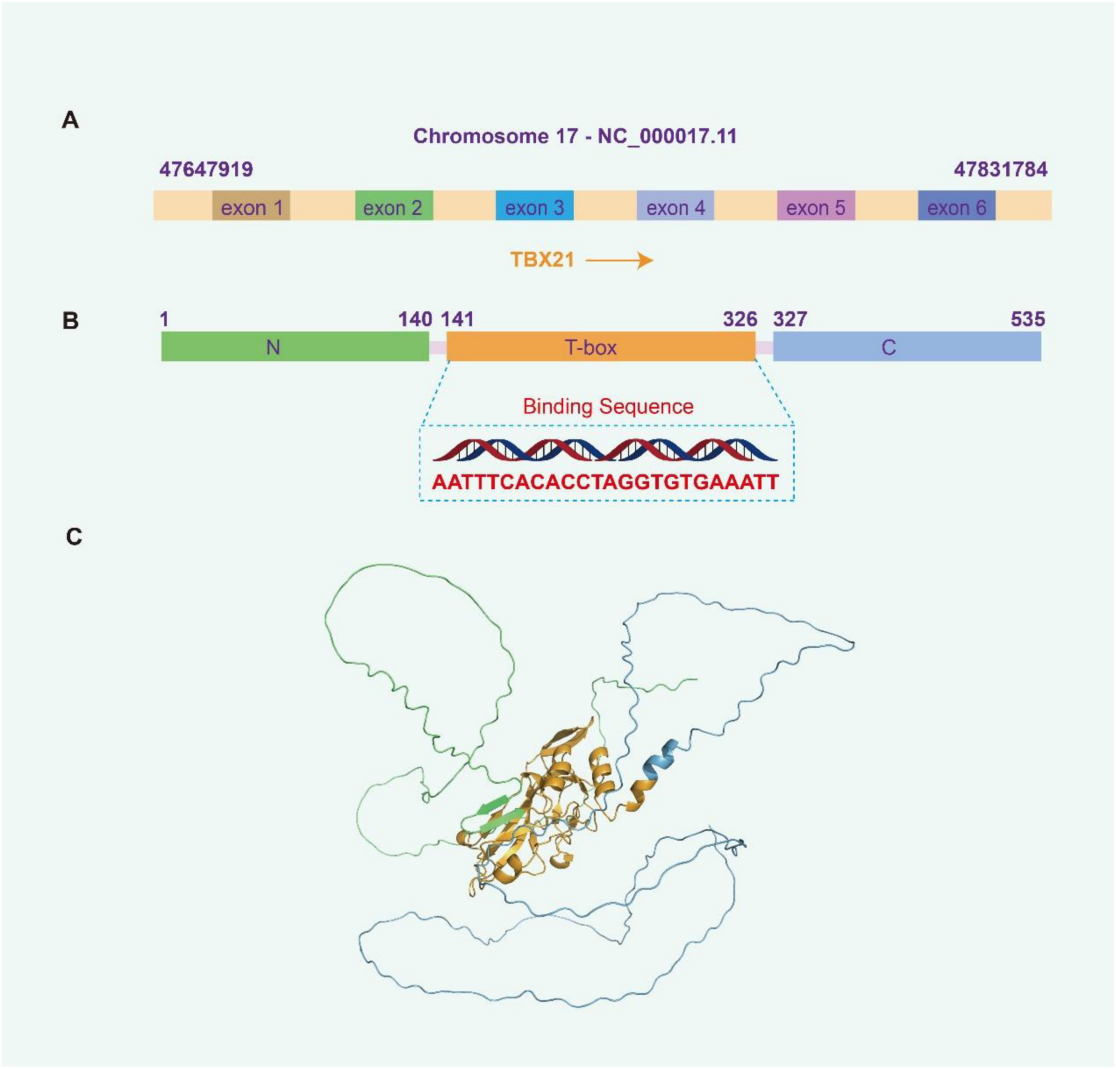
Basic information and structure of *TBX21*. **(A)** Location of the *TBX21* gene on the human chromosome (NCBI database). **(B)** Information on different structural domains of T-bet protein (Uniprot database), N-terminal domain: 1–140 amino acids (AA), T box domain: 141–326 AA, C-terminal domain: 327–535 AA. **(C)** Structure of T-bet. Green: N-terminal domain, yellow: T box domain, blue: C-terminal domain.

The transcription factor T-bet, encoded by the T-Box transcription factor 21 (*TBX21*) gene, functions as an immune cell-specific transcriptional regulator within the T-box family. This family is well-recognized for its essential involvement in the differentiation and functional modulation of various immune cell types. The *TBX21* gene is situated at the 17q21.32 locus on the human genome and is classified within the T-box subfamily Tbr1 (1). Detailed analyses reveal that the human *TBX21* gene encompasses approximately 10,000 base pairs and consists of multiple exons and introns (**Figure 1A**). Upon transcription and subsequent splicing, the mature mRNA is translated into the T-bet protein, which comprises 535 amino acid residues.

Structurally, the N-terminal region of T-bet spans roughly the first 100 to 150 amino acids and contains a transcriptional activation domain enriched with charged residues, including lysine and arginine. These charged amino acids are critical for mediating interactions with transcriptional coactivators and components of the RNA polymerase complex, thereby facilitating the transcriptional activation of target genes. The central portion of the protein harbors a highly conserved T-box domain, approximately 180 to 200 amino acids in length, characterized by a distinctive three-dimensional architecture. This domain consists of a series of α-helices and β-sheets arranged in a specific conformation that enables precise recognition and binding to the T-box DNA consensus sequence AATTTCACACCTAGGTGTGAAATT located within the promoter regions of target genes (2) (**Figure 1B**). The side-chain properties of amino acids within this domain are optimized to support DNA-protein interactions: hydrophobic residues contribute to maintaining the structural integrity of the protein, while polar residues engage directly with DNA bases. The C-terminal region, encompassing approximately 150 to 200 amino acids, also plays a significant role, potentially mediating interactions with other transcription factors or regulatory proteins. Specific residues within this region may be subject to post-translational modifications, such as phosphorylation, which can further modulate the functional activity of T-bet. As a principal transcription factor, T-bet predominantly localizes to the nucleus, where it orchestrates the regulation of gene expression (**Figures 1B, C**).

T-bet was initially identified through efforts aimed at isolating transcription factors responsible for the tissue-specific expression of Th1 cytokines (3). It is well-established that T-bet activates the expression of the Th1 signature cytokine IFN-γ while concurrently repressing IL-4 production in developing Th2 cells, thereby promoting the differentiation of naive T helper precursor cells toward the Th1 lineage (4). The expression of T-bet is induced by various cytokines, including IFN-γ, IL-12, IL-15, and IL-21, through their respective receptors and downstream signaling cascades such as JAK/STAT, PI3K-AKT-mTORC1, and TLR/MyD88 pathways (5). Although the regulatory role of T-bet in balancing immune responses and autoimmunity is complex, recent investigations have definitively identified T-bet as a pivotal regulator of type 1 proinflammatory immune responses across both adaptive and innate immune compartments (6). Moreover, contemporary research has expanded the known expression profile of TBX21 to include diverse immune cell populations such as B cells, dendritic cells, natural killer cells, and innate lymphoid cells, as well as non-immune cells including epithelial cells of the reproductive and respiratory tracts (7), terminal Schwann cells at the neuromuscular junction (8), and the olfactory bulb and thymus in murine models (9).

## 2 Biological functions of T-bet

### 2.1 T-bet is associated with the development of multiple immune cells

#### 2.1.1 T-bet and Th1 cell

**Figure 2:**
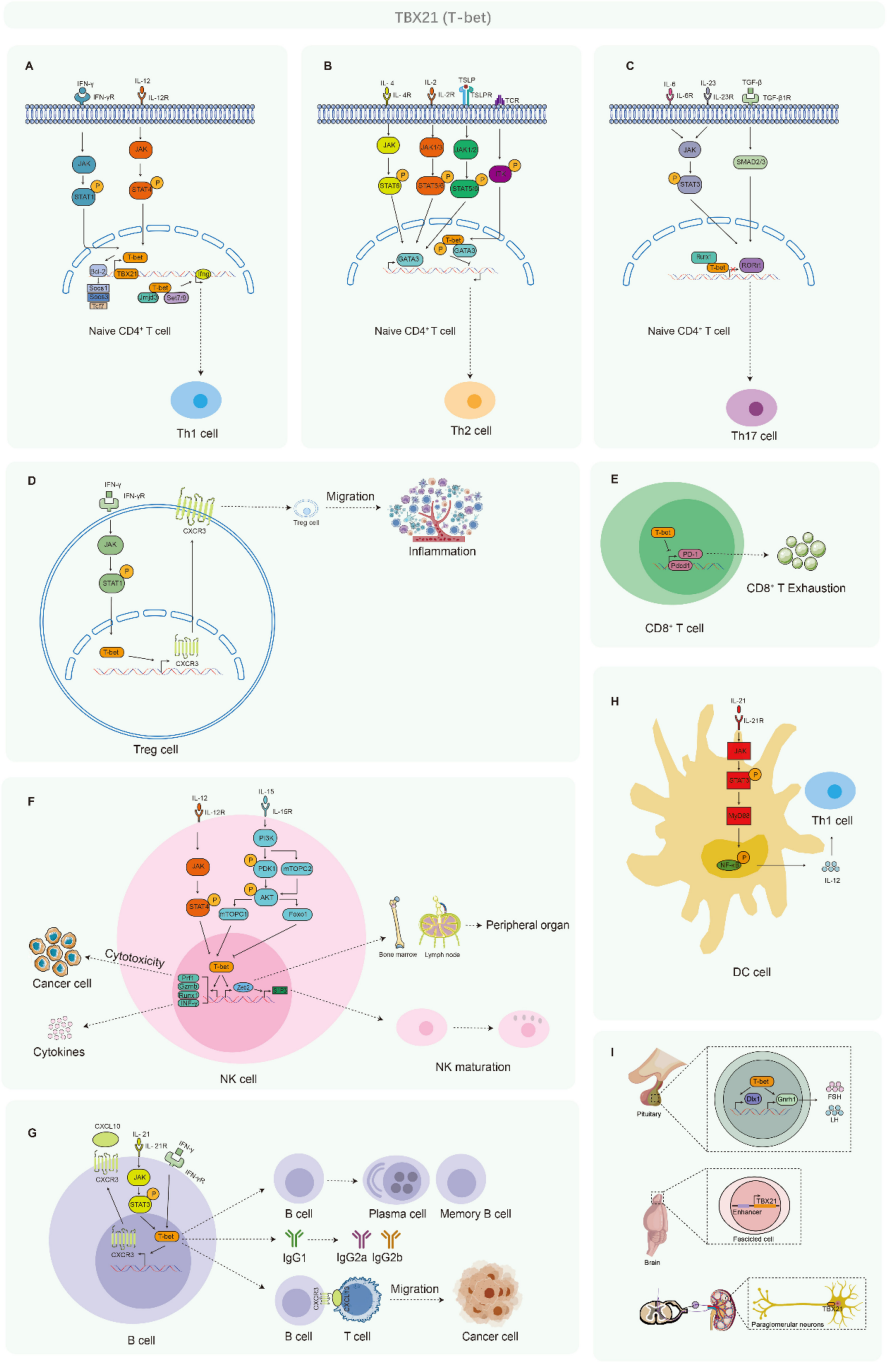
T-bet is associated with the development of multiple immune cells. **(A)** Mechanism of T-bet activation and guidance of Th1 lineage development. **(B)** Mechanism of T-bet activation and inhibition of Th2 lineage development. **(C)** Mechanism of T-bet activation and inhibition of Th17 lineage development. **(D)** The regulatory functions of T-bet in Treg cell activity. **(E)** The influence of T-bet on the exhaustion of CD8^+^ T cells. **(F)** The role of T-bet in the development of NK cells. **(G)** The regulatory functions and mechanisms of T-bet in B-cell activities. **(H)** The roles of T-bet in DCs. **(I)** The roles of T-bet in various other tissues or cell types.

T-bet functions as a lineage-defining transcription factor essential for the differentiation of Th1 cells and modulates Th1-specific gene expression by modifying the epigenetic or chromatin landscape of helper T cells (10). Cytokines, including IFN-γ and IL-12, promote the expression of T-bet through their respective receptors and associated downstream signaling pathways, such as JAK/STATs. T-bet facilitates the recruitment of chromatin remodeling complexes, which include histone H3K4 methyltransferases Set7/9 and histone H3K27 demethylase Jmjd3, to specific target genes, thereby inducing transcription, regulating Th1-specific gene expression, and enabling the binding of additional transcriptional activators, such as NFAT, AP-1, STAT4, and NF-κB (11). Furthermore, T-bet directly recruits the transcriptional repressor Bcl-6 to the promoter regions of certain genes, including Socs1, Socs3, and Tcf7, thereby inhibiting their expression and influencing Th1 lineage development (12). Additionally, T-bet engages in physical interactions with various regulatory factors, such as Runx3, which aids in its recruitment to gene loci (13) (**Figure 2A**).

#### 2.1.2 T-bet and Th2 cell

The differentiation of Th2 cells is contingent upon the expression of the transcription factor GATA3, which is predominantly regulated by cytokines including IL-4, IL-2, and TSLP. IL-4 facilitates the activation of the JAK/STAT6 signaling cascade via the IL-4Rα/γc, thereby promoting GATA3 transcription and the subsequent activation of Th2-specific genes (14, 15). Concurrently, IL-2 and TSLP contribute significantly to the proliferation and maintenance of Th2 cells by engaging their respective receptors, IL-2R and TSLPR, and initiating downstream signaling pathways that favor Th2 polarization (16, 17). In contrast, T-bet, recognized as the principal transcriptional regulator of the Th1 lineage, interacts with GATA3 to inhibit its capacity to activate Th2-associated genes. TCR signaling induces phosphorylation of T-bet by the kinase Itk, which enhances the T-bet–GATA3 interaction, thereby disrupting GATA3 function (18). This interaction not only impedes the transcriptional activation of Th2 genes through competitive binding mechanisms but also suppresses Th2 lineage programming via epigenetic modifications (19, 20). Consequently, while IL-4, IL-2, and TSLP facilitate Th2 differentiation by augmenting GATA3 activity, T-bet exerts an antagonistic effect on the Th1/Th2 lineage commitment by inhibiting GATA3 function (**Figure 2B**).

#### 2.1.3 T-bet and Th17 cell

The development of Th17 cells is contingent upon the transcription factor Rorγt, which is induced by cytokines such as IL-6 and IL-23. These cytokines activate their corresponding receptors, subsequently engaging the downstream JAK/STAT signaling pathways that promote the expression of Rorγt, thus facilitating Th17 cell differentiation. Conversely, T-bet exerts an inhibitory effect on the Th17 genetic program by forming a physical interaction with RUNX1, which obstructs the transcriptional activation of Rorc, the gene responsible for encoding Rorγt (21) (**Figure 2C**).

#### 2.1.4 T-bet and Treg cell

T-bet is a critical regulator of T regulatory (Treg) cell homeostasis and facilitates the accumulation of T-bet^+^ Treg cells at sites of Th1-mediated inflammation by promoting the expression of the chemokine receptor CXCR3 on Treg cells (22). Tregs that express T-bet are instrumental in modulating inflammatory responses. These Tregs adopt a Th1-like phenotype, which is characterized by the expression of CXCR3, IFN-γ, T-bet, and IL-10, a phenotype that is frequently observed in various parasitic infections. Additionally, Th1-like Treg cells have been identified in the context of viral infections, including those caused by lymphocytic choriomeningitis virus and influenza A. Notably, CXCR3^+^ T-bet^+^ IFN-γ^+^ Tregs are present in the spleen and lungs of infected hosts (23) (**Figure 2D**).

#### 2.1.5 T-bet and CD8^+^ T cell

T-bet exerts a direct inhibitory effect on the transcription of *PDCD1*, the gene responsible for encoding PD-1, in CD8^+^ T cells (24). This transcriptional regulation influences the depletion of CD8^+^ T cells and improves the management of viral replication in the context of chronic infections. CD8^+^ T cells exhibiting elevated levels of T-bet are predisposed to differentiate into terminally differentiated effector cells, in contrast to those with lower T-bet levels, which demonstrate an increased capacity for the development of memory cells (**Figure 2E**).

#### 2.1.6 T-bet and NK cell

In NK cells, IL-12 engages the IL-12R to activate the JAK2/STAT4 signaling cascade, which subsequently upregulates T-bet expression, thereby augmenting NK cell-mediated immune surveillance against tumor cells (25). Similarly, IL-15 stimulates the PI3K/AKT/mTORC1 signaling pathway through its receptor IL-15R, leading to increased T-bet expression and the enhancement of NK cell effector functions, including IFN-γ secretion and cytotoxic activity (26, 27).

T-bet collaborates with Eomesodermin (Eomes) to facilitate the development of NK cells, and T-bet is essential for the maturation of these cells (28). The downregulation of T-bet has been identified as a marker indicative of NK cell exhaustion within the tumor microenvironment. T-bet enhances the transcription of several genes, including *Prf1*, *Gzmb*, and *Runx1*, which are critical for mediating NK cell cytotoxicity. Furthermore, T-bet promotes the expression of *Zeb2*, which is instrumental in the differentiation of immature NK cells into their mature counterparts. T-bet induces *Zeb2*, which subsequently binds to the promoter of *S1pr5*, facilitating the egress of mature NK cells from lymphoid tissues and bone marrow to peripheral organs. The T-bet/Eomes-IFN-γ signaling axis in NK cells is vital for the control of tumors dependent on CD8^+^ T cells, and the effector functions of NK cells that are reliant on T-bet are crucial for achieving optimal responses to anti-PD-L1 immunotherapy. Additionally, the absence of *Tipe2* in NK cells not only enhances their intrinsic anti-tumor capabilities but also indirectly bolsters the anti-tumor response of CD8^+^ T cells by promoting T-bet/Eomes-dependent NK cell effector functions (29) (**Figure 2F**).

#### 2.1.7 T-bet and B cell

In B lymphocytes, IL-21 engages its receptor, IL-21R, leading to the activation of the Janus kinases JAK1 and JAK3. This activation subsequently triggers the phosphorylation and activation of the transcription factor STAT3. Once activated, STAT3 translocated into the nucleus, where it binds to the promoter region of the T-bet gene, thereby upregulating T-bet expression (30). This molecular cascade promotes the differentiation of B cells toward a Th1-type immune response and augments the production of IFN-γ.

T-bet functions as a selective inducer of the transcriptional expression of *IgG2α* and facilitates IFN-γ-mediated class switching in B cells (31). The IFN-γ/T-bet-dependent signaling pathway modulates the expression of CXCR3 in B cells, thereby promoting the migration of memory B cells to sites of inflammation (32). Expansion of T-bet^+^ B cells is characterized by the absence of complement receptors CD21 and CD27, alongside an increased expression of CD11c. Elevated levels of T-bet expression in B cell subsets have been observed in the circulation of individuals suffering from various chronic conditions, including sepsis, chronic infections such as HIV, coronavirus disease, malaria, and hepatitis, as well as autoimmune disorders like systemic lupus erythematosus (SLE), primary Sjögren’s syndrome, rheumatoid arthritis, juvenile idiopathic arthritis, common variable immunodeficiency, and neuromyelitis optica spectrum disorders (33). Furthermore, studies in autoimmune mouse models have indicated that T-bet^+^ B cells and their progeny serve as precursors for the production of autoantibodies (34), suggesting that T-bet expression in B cells or related cell types may influence disease progression (**Figure 2G**).

#### 2.1.8 T-bet and DCs

T-bet is expressed in DCs at levels similar to those observed in Th1 cells, and it plays a critical role in the production of IFN-γ as well as in the activation of antigen-specific Th1 cells (35). In innate immune cells, including DCs, PAMPs initiate TLR-mediated signaling pathways that depend on the adaptor protein MyD88. This signaling cascade facilitates the production of Th1-type cytokines, such as IL-12 (36), by activating transcription factors including NF-κB, and subsequently upregulates T-bet expression indirectly. Through the secretion of IL-12, dendritic cells play a critical role in promoting the polarization of T cells toward the Th1 phenotype (37, 38) (**Figure 2H**).

#### 2.1.9 T-bet and ILCs

Innate lymphoid cells (ILCs) represent a category of innate lymphocytes characterized by the absence of RAG-dependent rearranged antigen-specific cell surface receptors. They are classified into five distinct types: NK cells, which exhibit cytotoxic properties, and helper-like ILCs, which include ILC1, ILC2, and ILC3. The functional roles of these helper-like ILCs correspond to those of CD4^+^ T helper (Th) cells, specifically Th1, Th2, and Th17 cells, respectively, as well as lymphoid tissue inducer (LTi) cells. The differentiation of ILC1 is predominantly governed by the transcription factor T-bet, while GATA3 regulates the ILC2 lineage, and the RORγt is responsible for the ILC3 lineage (39). Notably, ILC2s have the capacity to transdifferentiate into ILC1s under conditions of chronic inflammation, a phenomenon observed in patients suffering from chronic obstructive pulmonary disease and Crohn’s disease (40–43). In such inflammatory contexts, the presence of IL-1β and IL-12 leads to a downregulation of GATA3 in ILC2s, concomitantly resulting in an upregulation of T-bet and IFN-γ production.

### 2.2 The function of T-bet in other tissues or cells

T-bet displays multifaceted functions extending beyond the immune system, notably within neural tissues where it modulates reproductive processes through the regulation of Gnrh1 expression in the hypothalamus. Additionally, it shows distinct expression profiles in neurons of the olfactory bulb. These observations underscore the pleiotropic roles of T-bet in mammalian development and physiological regulation. Xiaoning Li and colleagues have demonstrated that T-bet functions as a transcriptional activator that directly enhances the expression of gonadotropin-releasing hormone 1 (Gnrh1) by binding to its promoter region. Additionally, T-bet indirectly promotes Gnrh1 expression through the activation of another transcriptional activator, Dlx1. The research revealed that female mice exhibiting ectopic expression of *Tbx21* in the hypothalamus experienced alterations in both the onset of puberty and reproductive capacity. Specifically, these mice displayed elevated serum levels of luteinizing hormone (LH) and follicle-stimulating hormone (FSH), larger litter sizes, but a more pronounced decline in reproductive capacity compared to the control group (44). Consequently, miR-29-3p and its target *TBX21* are implicated in the regulation of puberty onset and reproductive functions in mammals through the modulation of Gnrh1 expression. Furthermore, Sachiko Mitsui and collaborators identified that *Tbx21* is specifically expressed in the mitral and tufted cells of the olfactory bulb. They found that cis-regulatory enhancer elements, approximately 300 nucleotides in length and located about 3.0 kb upstream of the *Tbx21* gene transcription start site, facilitate transgene expression in these cells. In contrast, the 2.6 kb 5’-flanking region of the mouse *Tbx21* gene induces transgene expression in a variable manner across restricted neuronal populations primarily situated within the olfactory pathway (45). Additionally, the intensity of immune reactivity staining for *Tbx21* exhibits significant variability among different subgroups of periglomerular neurons, thereby allowing *Tbx21* to serve as a further distinguishing marker for periglomerular neurons in the main olfactory bulb of mice (46) (**Figure 2I**).

## 3 *TBX21* SNP and disease occurrence

**Figure 3:**
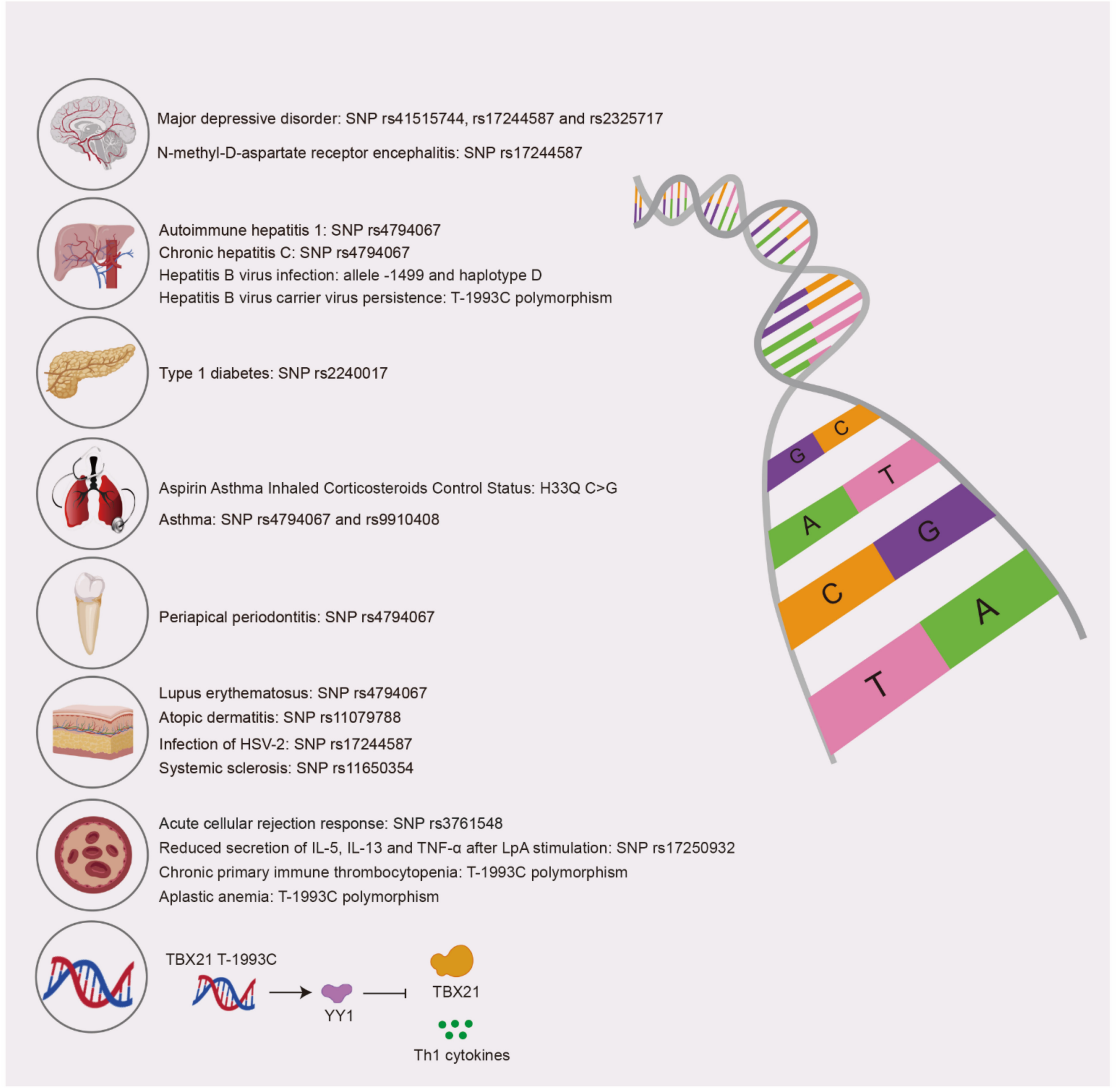
The relationship between single nucleotide polymorphisms (SNPs) in the *TBX21* gene and the incidence of disease.

The human *TBX21* gene is characterized by 40 identified polymorphisms. Notably, SNP rs2240017 has been linked to an increased susceptibility to type 1 diabetes (47). Additionally, *TBX21* SNP rs17244587 has been associated with the incidence of herpes simplex virus type 2 (HSV-2) (48). Other polymorphisms, specifically rs4794067, rs2275806, rs2232365, and rs3761548, are situated within the *TBX21*, *GATA3*, and *FOXP3* genes, which play a role in mediating acute cellular rejection responses. Research conducted by Hansjörg Thude et al. indicates that the -3279A allele of SNP rs3761548 may increase the risk of developing late acute cellular rejection (49). Furthermore, Vera Isabel Casaca and colleagues discovered that individuals carrying the *TBX21* promoter SNP rs17250932 and the *HLX1* promoter SNP rs2738751 exhibited reduced or trending reductions in the secretion of IL-5, IL-13, and TNF-α following LpA stimulation. Additionally, carriers of the *TBX21* SNP rs11079788 reported fewer symptoms of atopic dermatitis by the age of three. The polymorphisms within *TBX21* and *HLX1* primarily influence the secretion of IL-5 and IL-13 in cord blood post-LpA stimulation, suggesting that genetic variations in transcription factors critical for the Th1 pathway may play a role in modulating Th2 immune responses during early life (50) (**Figure 3**).

The SNP of the *TBX21* gene influences the expression levels of T-bet and is linked to susceptibility to various diseases. For instance, the rs4794067 variant of *TBX21* is correlated with an increased risk of severe asthma, lupus, type 1 autoimmune hepatitis, and periapical periodontitis (51), (52). Additionally, the *TBX21* rs9910408 variant (53) has been associated with treatment outcomes for asthma in adults, while the *TBX21* H33Q C>G variant shows a significant relationship with inhaled corticosteroid control status in patients with aspirin-induced asthma (54). Polymorphisms such as rs41515744, rs17244587, and rs2325717 have been linked to the phenotype of major depressive disorder (MDD) (55). Furthermore, the *TBX21* rs172445874 (56) variant is associated with anti-N-methyl-D-aspartate receptor (NMDAR) encephalitis. The SNP rs11650354 of *TBX21* has been found to be associated with systemic sclerosis (57). Moreover, copy number variations in *TBX21* have been implicated in susceptibility to ankylosing spondylitis and acute anterior uveitis (51). The C allele of rs4794067 in the *TBX21* promoter is more prevalent among patients with chronic hepatitis C (58), suggesting a potential role of *TBX21* variation in the etiology of this condition. Genetic variants, including allele -1499 and haplotype D (--/AC) in the *TBX21* promoter region, appear to increase susceptibility to hepatitis B virus (HBV) infection within a Chinese population (59). Additionally, research indicates that the T-1993C polymorphism in the *TBX21* promoter is associated with susceptibility to persistent HBV infection (60). The T-1993C polymorphism of the *TBX21* gene may also be linked to susceptibility to chronic primary immune thrombocytopenia and aplastic anemia in the Chinese population (61, 62), as well as to the persistent presence of the virus in HBV carriers (60) (**Figure 3**).

Furthermore, J-R Li et al. demonstrated that the *TBX21* promoter containing the -1993C allele exhibits a significantly greater binding affinity for the Yin Yang 1 (YY1) transcription factor in comparison to the promoter with the -1993T allele, with YY1 functioning as a repressive protein. The substitution of cytosine (C) with thymine (T) at this position mitigates the repressive effects mediated by YY1 (63). These results indicate that the *TBX21* T-1993C polymorphism may inhibit T-bet expression and the production of Th1 cytokines by modulating YY1 activity, potentially contributing to the dysregulation between Th1 and Th2 immune responses observed in autoimmune and allergic diseases (**Figure 3**).

## 4 T-bet and non-neoplastic diseases

**Figure 4:**
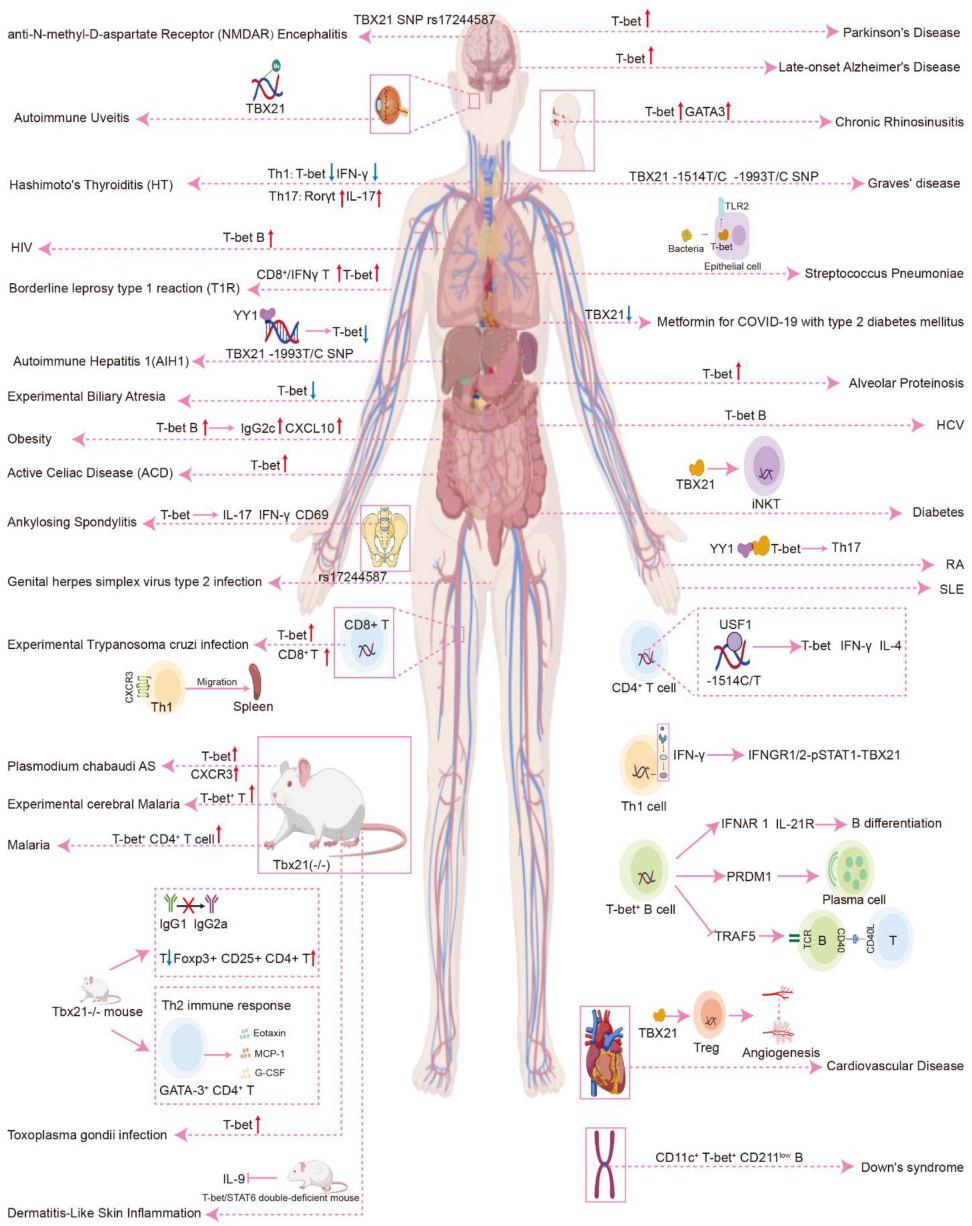
Role of T-bet in non-tumorigenic diseases.

This section provides an overview of the role of T-bet in the pathogenesis of various diseases across multiple bodily systems, encompassing autoimmune disorders, infectious diseases, allergic conditions, endocrine disorders, psychiatric illnesses, and chromosomal abnormalities. A comprehensive examination of the significant contributions of T-bet to the development of non-neoplastic diseases, along with the underlying pathogenic mechanisms, is presented in detail (**Figure 4**).

### 4.1 Autoimmune diseases

#### 4.1.1 Thyroiditis

The functional polymorphism of the *TBX21* gene is significantly linked to the development and clinical outcomes of autoimmune thyroid disorders, including Graves’ disease and Hashimoto’s thyroiditis. Variations in its expression play a critical role in the pathogenesis of these conditions by modulating the dysregulation of Th1 and Th17 cell differentiation. Research conducted by Mami Morita and colleagues has demonstrated that the T alleles of the *TBX21*-1514T/C and -1993T/C polymorphisms are more prevalent in individuals diagnosed with refractory Graves’ disease (GD) compared to those in remission (64). This suggests that functional polymorphisms in *TBX21* are linked to the development of autoimmune thyroid diseases and the prognosis of GD. Furthermore, *TBX21* is also significantly associated with the onset of Hashimoto’s thyroiditis (HT). In patients suffering from HT, there is a notable decrease in the expression levels of *TBX21* and *IFN-γ* mRNA, which are markers associated with Th1 cells, in peripheral mononuclear cells. Conversely, the mRNA levels of *Rorγt* and *IL-17*, which are associated with Th17 cells, are elevated in these patients (65). These findings imply that the dysregulation of Th1/Th17 differentiation in peripheral blood mononuclear cells may play a role in the pathogenesis of Hashimoto’s thyroiditis. Additional studies have indicated that severe forms of HT, rather than hyperthyroid HT, are correlated with a significant upregulation of *TBX21* and *FOXP3* mRNA in peripheral T cells, a phenomenon that appears to be independent of thyroid hormone levels but is proportional to the activity of the disease (66).

#### 4.1.2 Active celiac disease

In the context of intestinal mucosal immunity, *TBX21* has been implicated in pathologies arising from the dysregulation of intestinal immune cell populations, particularly in relation to the polarization of Th1 and Th17 cells. Research conducted by Fatemeh Ganjali and colleagues demonstrated that, in comparison to a control group, the mRNA expression levels of *TBX21* were significantly elevated in individuals diagnosed with active celiac disease (67).

#### 4.1.3 Autoimmune hepatitis

Polymorphisms in the *TBX21* gene are associated with the development of type 1 autoimmune hepatitis. Research has demonstrated that the T-1993C polymorphism within the promoter region of the *TBX21* gene influences the susceptibility of the Chinese population to type 1 autoimmune hepatitis (AIH-1) (68). Wei Sun and colleagues have identified that YY1 exhibits a strong binding affinity for the -1993C allele, which in turn inhibits the expression of *TBX21*. This inhibition may contribute to a decreased incidence of AIH-1 by attenuating type 1 immune responses (69).

#### 4.1.4 Autoimmune encephalitis

*TBX21* is closely associated with the pathogenesis of autoimmune encephalitis. Y Shu et al. identified a notable association between NMDAR encephalitis and the *TBX21* rs17244587 polymorphism. The relationships observed among *IRF7*, *BANK1*, and *TBX21* in the context of anti-NMDAR encephalitis imply that mechanisms such as B-cell activation, the Th1 immune response, viral infections, and the type I interferon signaling pathway may play a role in the pathogenesis of this condition (56).

#### 4.1.5 Systemic lupus erythematosus

Promoter polymorphisms of *TBX21*, such as T-1514C, are strongly associated with susceptibility to SLE and contribute to disease pathogenesis by regulating Th1 cytokine production and B-cell dysfunction. Junggang Li et al. identified two SNPs, T-1993C and T-1514C, located in the promoter region of the *TBX21* gene, which are associated with SLE. The T-1514C polymorphism has been shown to influence *TBX21* gene expression and the production of Th1 cytokines by facilitating the binding of USF-1 to the SNP site (70). Furthermore, Ying Chen and colleagues demonstrated that IFN-γ contributes to the pathogenesis of SLE through the IFNGR1/2-pSTAT1-T-bet signaling pathway, which modulates inflammation and the formation of immune complexes in murine models of SLE (71). Additional research has indicated that the interaction between IFNG and IFNG-T-bet is linked to susceptibility to SLE (72). T-bet^+^ B cells have been implicated in the development of SLE, exhibiting transcriptional profiles akin to activated naive B cells in lupus patients. These cells display heightened expression of type III IFN-γ receptor, IFN-λ receptor 1, and IL-21 receptor, which promotes the differentiation of naive B cells. Both T-bet^+^ B cells and activated naive B cells in lupus patients demonstrate downregulation of the gene TNF receptor-associated factor 5, a mediator of T: B interactions that regulates CD40 signaling and negatively modulates TLR signaling. Moreover, B cells expressing T-bet, along with activated immature B cells in lupus patients, show increased expression of the plasma cell-defining transcription factor PRDM1 and chromatin remodeling at the PRDM1 promoter, resulting in dysregulated humoral immune responses (33). CD27^+^ T-bet^+^ B cells enhance the expression of transcription factors IRF4 and PRDM1, which are critical for plasma cell differentiation, and sustain responsiveness to T cell help signals such as IL-21 and CD40 ligand.

#### 4.1.6 Rheumatoid arthritis, ankylosing spondylitis and autoimmune arthritis

T-bet plays a key role in the pathogenesis of autoimmune arthritis such as rheumatoid arthritis and ankylosing spondylitis. Jinpiao Lin and colleagues have identified that YY1 plays a regulatory role in the pathogenicity of Th17 cells by binding to the promoter region of the transcription factor T-bet and interacting with the T-bet protein, thus contributing to the pathogenesis of RA (73). Furthermore, TBX21 exhibits elevated expression levels in patients with AS, primarily influenced by natural killer and CD8^+^ T cells, which in turn enhances the expression of IL-17 and IFN-γ (74). This finding indicates that T-bet is a critical element of the inflammatory pathway in both human and murine spondyloarthropathies. Additionally, the researchers observed that interferon gamma can inhibit the differentiation of Th17 cells in a manner that is independent of T-bet expression in autoimmune arthritic mouse models (75).

#### 4.1.7 Systemic sclerosis

The genetic variant rs11650354 in *TBX21* is associated with susceptibility to systemic sclerosis and participates in immune dysregulation processes in multiple sclerosis. In a thorough investigation involving 902 patients with SSc and 4,745 control subjects, Pravitt Gourh et al. identified a recessive pattern of disease susceptibility associated with the TT genotype of the *TBX21* rs11650354 variant. Additionally, the A allele of the *STAT4* variant rs11889341 was found to correlate with heightened susceptibility to SSc within a dominant genetic model. Notably, the interaction between the *TBX21* and *STAT4* variants indicated that the *STAT4* genotype elevated the risk of SSc exclusively in individuals possessing the *TBX21* CC genotype. When compared to the control group, SSc patients with the *TBX21* CC genotype exhibited increased levels of IL-6 and tumor necrosis TNF-α, whereas those with the TT genotype demonstrated elevated levels of interleukins IL-2, IL-5, IL-4, and IL-13, indicative of a Th2 response. Whole blood expression profiles revealed that the CC genotype of the *TBX21* SNP was associated with a disrupted type I interferon signaling pathway, while the TT genotype was linked to alterations in T cell signaling pathways (57). Furthermore, the researchers observed that blood samples from patients with multiple sclerosis exhibited a higher frequency of IFN-γ^+^ regulatory T cells compared to healthy controls, which in turn facilitated the expression of pro-inflammatory genes, including *TBX21*, *IFN-g*, and *HIF-1α* (76) (77).

#### 4.1.8 Other autoimmune diseases

T-bet participates in immune dysregulation mechanisms by regulating Treg cell function in various autoimmune diseases such as uveitis, encephalomyelitis, and vitiligo. The research identified notable alterations in methylation patterns of *Tbx21* and *Rorc* at a CpG site within the retinas of mice subjected to experimental autoimmune uveitis (78). The role of T-bet in Treg cell is critical for modulating the progression of experimental autoimmune encephalomyelitis (79). Conversely, in distinct contexts, such as the nephrotoxic nephritis model associated with crescentic glomerulonephritis, T-bet in Tregs is crucial for regulating renal inflammation, as its absence hinders the migratory capacity of Tregs to the kidney (80). Furthermore, in peripheral blood mononuclear cells from individuals with vitiligo, there is an increased prevalence of circulating T-bet^+^ Treg cells, accompanied by a diminished suppressive function. Consequently, a potential clinical approach may involve targeting the TH1-skewed microenvironment present in serum to mitigate inflammation and enhance immunosuppression (81).

### 4.2 Infectious diseases

#### 4.2.1 Viral infection

*TBX21* plays a pivotal role in viral infections, with its expression levels and polymorphisms influencing disease progression and risk in chronic hepatitis C, HIV, and herpes simplex virus infections by regulating B-cell and T-cell function. HIV and chronic hepatitis C have been shown to enhance the expression of T-bet in B cells (82), thereby initiating an antiviral response within these cells. Notably, the rs4794067 C allele in the *TBX21* promoter is found to be significantly more prevalent among individuals diagnosed with chronic hepatitis C (58). In the context of HIV infection, some patients undergoing antiretroviral therapy exhibit low-level viremia (LLV), which is attributed to the peripheral blood CD4^+^ T cell pool. Research conducted by Jingliang Chen et al. indicates that CD4^+^ T cells associated with LLV demonstrate elevated expression levels of the Th1 signature transcription factor, *TBX21* (83). Furthermore, Stefan Petkov’s findings reveal that HIV-1-infected individuals receiving early antiretroviral therapy show a lack of regulation in 20 critical regulators of T cell differentiation, including T-bet, GATA3, RARA, FOXP3, and RORC, which leads to compromised differentiation of CD4^+^ T cells (84). Additionally, miR-31 has been identified as a direct inhibitor of the upstream *STAT1* transcription factor, which downregulates T-bet, consequently inhibiting the production of IFN-γ and the activation of CD4^+^ T cells, thus serving a protective function during HIV infection (85). Moreover, the overexpression of T-bet in the context of HIV infection correlates with the accumulation of B cells outside of germinal centers and results in suboptimal affinity maturation. Other investigations have identified polymorphisms in the 3’-untranslated region of the *TBX21* gene, which encodes T-bet, as potential risk factors for infection with human genital herpes simplex virus type 2 (48).

#### 4.2.2 Bacterial infection

T-bet participates in host defense mechanisms against bacterial infections such as tuberculosis, leprosy, and pneumonia by regulating immune cell function and inflammatory responses. Julio Flores-Gonzalez and colleagues identified the expression of granzyme A, granzyme B, granulysin, and perforin, along with the genes T-bet and NKG2D, in enriched CD4^+^ T cells, thereby confirming their cytotoxic capabilities (86). The presence of CD4^+^ T cells is indicative of active tuberculosis. Furthermore, Mycobacterium tuberculosis has been shown to stimulate the production of T-bet^+^ Treg cells, which play a crucial role in modulating inflammation within tissues. A strong correlation was observed between T-bet expression and the frequency of CD8^+^/IFN-γ T cells, which is associated with the development of a borderline leprosy type 1 response (T1R) (87). Notably, T-bet expression was significantly elevated in T1R cases. Additionally, T-bet has been linked to pneumococcal pneumonia. Research conducted by C. H. Woo and colleagues demonstrated that pneumococcus enhances the expression of T-bet in respiratory epithelial cells and contributes to the regulation of the innate immune response by modulating TLR2 expression (7).

#### 4.2.3 Parasitic infection

T-bet influences host immune protection and disease progression in parasitic infections such as malaria and toxoplasmosis by regulating Th1/Th2 balance, antibody responses, and regulatory T cell function. T-bet plays a crucial role in modulating antibody responses and providing immunoprotection during malaria infections in murine models (88). Specifically, infection with Plasmodium yoelii 17XNL leads to a significant proliferation of activated Th1 (CD69 T-bet) CD4^+^ T cells, while the activation of T-bet CD8^+^ T cells remains comparatively low. Mice deficient in *Tbx21* (T-bet) exhibited a marked decrease in parasite burden, which was correlated with elevated levels of IgG1. This observation suggests that T-bet-mediated isotype switching of antibodies may contribute to the observed reduction in parasitic load. Furthermore, the absence of T-bet was associated with a temporary yet notable decline in T cell populations during the course of infection, indicating that T-bet may play a role in inhibiting malaria-induced apoptosis or promoting T cell proliferation. Conversely, *Tbx21*^-/-^ mice demonstrated an increased production of Foxp3 CD25 regulatory CD4 T cells, which may facilitate the early contraction of T cell populations (88). Additionally, the research indicated an increase in CD4 T-bet Foxp3 T cells in B6 mice during acute infection. The study also noted a significant rise in T-bet Foxp3 regulatory T cells, which exhibited a high frequency of CXCR3 expression. The induction of Th1-like CD4 T-bet Foxp3 regulatory T cells expressing CXCR3 during the acute blood stage of malaria suggests that CXCR3 expression on CD4 Th1 cells may enhance their migration to the spleen. The pathogenesis of experimental cerebral malaria (ECM) is, in part, an immune-mediated process involving Th1 CD4^+^ T cells. Investigations utilizing the Plasmodium berghei ANKA mouse model of ECM, alongside mice lacking the transcription factor T-bet on a susceptible C57BL/6 background, revealed that while T-bet is instrumental in regulating parasite load, it also plays a significant role in the pathogenesis of ECM. In the absence of T-bet, there was an enhancement of the Th2 immune response, characterized by an increased production of activated GATA-3^+^ CD4^+^ T cells and elevated levels of eosinophil chemokines, as well as MCP-1 and G-CSF cytokines (89).

The expression of T-bet in Treg is essential for mitigating Th1-mediated immunopathology during Toxoplasma gondii infection in murine models (90). Following a lethal oral challenge with Toxoplasma gondii (91), Tregs adopt a Th1-like phenotype, which is characterized by the expression of CXCR3, IFN-γ, T-bet, and IL-10. During acute Toxoplasma gondii infections, the presence of T-bet in Tregs is critical for the survival of the host. The absence of T-bet in Tregs hinders their migration to the lamina propria of the small intestine. Additionally, infection with Listeria monocytogenes prompts the *de novo* differentiation of Tregs into T-bet^+^ Tregs. Similarly, infection with Salmonella enterica (92) can also stimulate the generation of T-bet^+^ Tregs, which play a role in controlling tissue inflammation. Moreover, in the context of experimental Trypanosoma cruzi infection, T-bet enhances immunity to Trypanosoma cruzi by facilitating the expansion of Trypanosoma cruzi-specific CD8^+^ T cells through a T cell-intrinsic mechanism (93).

### 4.3 Allergic diseases

T-bet plays a crucial role in allergic diseases such as asthma, allergic rhinitis, and atopic dermatitis by regulating the Th1/Th2 cell balance and inflammatory response. A total of ten genes, including *TBX21*, have been recognized as central genes associated with childhood asthma (94). Research conducted by Huijuan Ma et al. has demonstrated that T-bet is significantly involved in asthma induced by formaldehyde, with findings indicating that formaldehyde exacerbates inflammatory responses and skews T helper cell lineage differentiation via the IFN-γ/STAT1/T-bet signaling pathway in the context of asthma (95). Furthermore, T-bet plays a crucial role in the management of asthma. Sodium fisetate has been shown to modulate the balance between Th1 and Th2 cells by enhancing the mRNA and protein expression of T-bet in the lung tissues of asthmatic mice, while concurrently down-regulating *GATA3*, thus facilitating Th1 cell differentiation and mitigating ovalbumin-induced pathological alterations in the lungs of these mice (96). Additionally, quercetin has emerged as a promising anti-asthmatic compound, as it has been found to decrease the expression of *Gata3, Tnf, Tgfb1, Il1b*, and *Acta2* genes, while increasing the expression of the *Tbx21* gene, thereby alleviating oxidative stress and inflammation associated with asthma in rat models (97). Treatment with Hanchuan Zupa Granule in a guinea pig model of cough-variant asthma resulted in elevated mRNA and protein levels of T-bet, alongside reduced mRNA and protein levels of TLR4 and GATA3, effectively alleviating the symptoms of cough-variant asthma by correcting the Th1/Th2 imbalance and modulating TLR4 receptor activity (98).

Furthermore, T-bet has been identified as a tissue biomarker of chronic sinusitis, exhibiting a correlation with eosinophil peroxidase and GATA3 (99). Additionally, T-bet and STAT6 work in concert to inhibit the production of IL-9 in CD4 T cells that is dependent on thymic stromal lymphopoietin, consequently mitigating skin inflammation resembling atopic dermatitis (100).

### 4.4 Neurodegenerative disease

T-bet is upregulated in neurodegenerative diseases such as Alzheimer’s disease and Parkinson’s disease, suggesting it may participate in disease progression by regulating peripheral immunity. S. R. Fatemi Langroudi and colleagues observed that the expression levels of T-bet were markedly increased in peripheral blood leukocytes of individuals diagnosed with late-onset Alzheimer’s disease. Furthermore, they noted a tendency for T-bet expression to rise with advancing age in patients with LOAD when compared to control subjects (101). Additionally, T-bet is found to be highly expressed in individuals suffering from idiopathic rapid eye movement sleep behavior disorder, as well as in patients with Parkinson’s disease, where it plays a role in the dysregulation of CD4^+^ T cells associated with the disease (102).

### 4.5 Down’s syndrome

T-bet plays a crucial role in immune dysregulation associated with Down’s syndrome. Malle et al. conducted a study to analyze both soluble and cellular immune responses in individuals with Down syndrome. Their findings revealed the consistent elevation of up to 22 cytokines at steady state, indicating a baseline level of cellular activation. Notably, they observed chronic IL-6 signaling in CD4^+^ T cells, alongside an increased prevalence of plasma cells and CD11c^+^ T-bet^+^ CD211^low^ B cells. Furthermore, the researchers identified that the frequency of CD11c^+^ T-bet^+^ CD211^low^ B cells serves as a potential marker for immune dysregulation associated with Down syndrome (103).

### 4.6 Diabetes

Genetic variations in *TBX21* contribute to the development of type 1 diabetes and influence disease progression and treatment response by regulating Th1 cell and immune cell function. The Gln-positive phenotype and (CA)14 allele in 3'-flanking region of T-bet were found to be associated with type 1 diabetes (47). T-bet is known to influence the pathogenesis of diabetes by modulating the production of IFN-γ in Th1 cells. Research conducted by Pavlo Petakh et al. demonstrated that administering metformin to COVID-19 patients with type 2 diabetes resulted in an increased diversity of the intestinal microbiota and a down-regulation of T-bet expression (104). Additionally, T-bet is implicated in the reprogramming of invariant natural killer T (iNKT) cells residing in the liver, which serves to suppress autoimmunity (105) related to hepatic function and diabetes. The conditional ablation of T-bet in Treg cells in *Foxp3^cre-^* deficient mice has been shown to lead to the onset of diabetes and its exacerbation (106).

### 4.7 Other diseases

T-bet is involved in regulating various components of the immune response and has been linked to the onset of systemic multisystem diseases. Research by Thomas Hägglöf and colleagues demonstrated that in cases of progressive obesity, T-bet^+^ B cells accumulate in adipose tissue, promoting the production of IgG2c and CXCL10, which worsens metabolic issues and pancreatic damage associated with obesity (107). Additionally, Sujit K Mohanty and his team discovered that a lack of T-Bet reduced bile duct injury in a model of experimental biliary atresia. In the context of cardiovascular disease, T-bet has been shown to activate regulatory T cells and promote angiogenesis, helping to reduce inflammation and replace damaged blood vessels (108). Furthermore, excessive expression of T-bet in T-lymphocytes results in the maturation of mononuclear phagocyte lineage cells and leads to severe secondary alveolar proteinosis (109).

Furthermore, it has been demonstrated that T-bet is instrumental in preserving tumor stemness and significantly contributes to tumor proliferation, apoptosis, and metastasis (110), as elaborated upon in the subsequent sections.

## 5 TBX21 and neoplastic diseases

### 5.1 Gastric cancer

**Figure 5:**
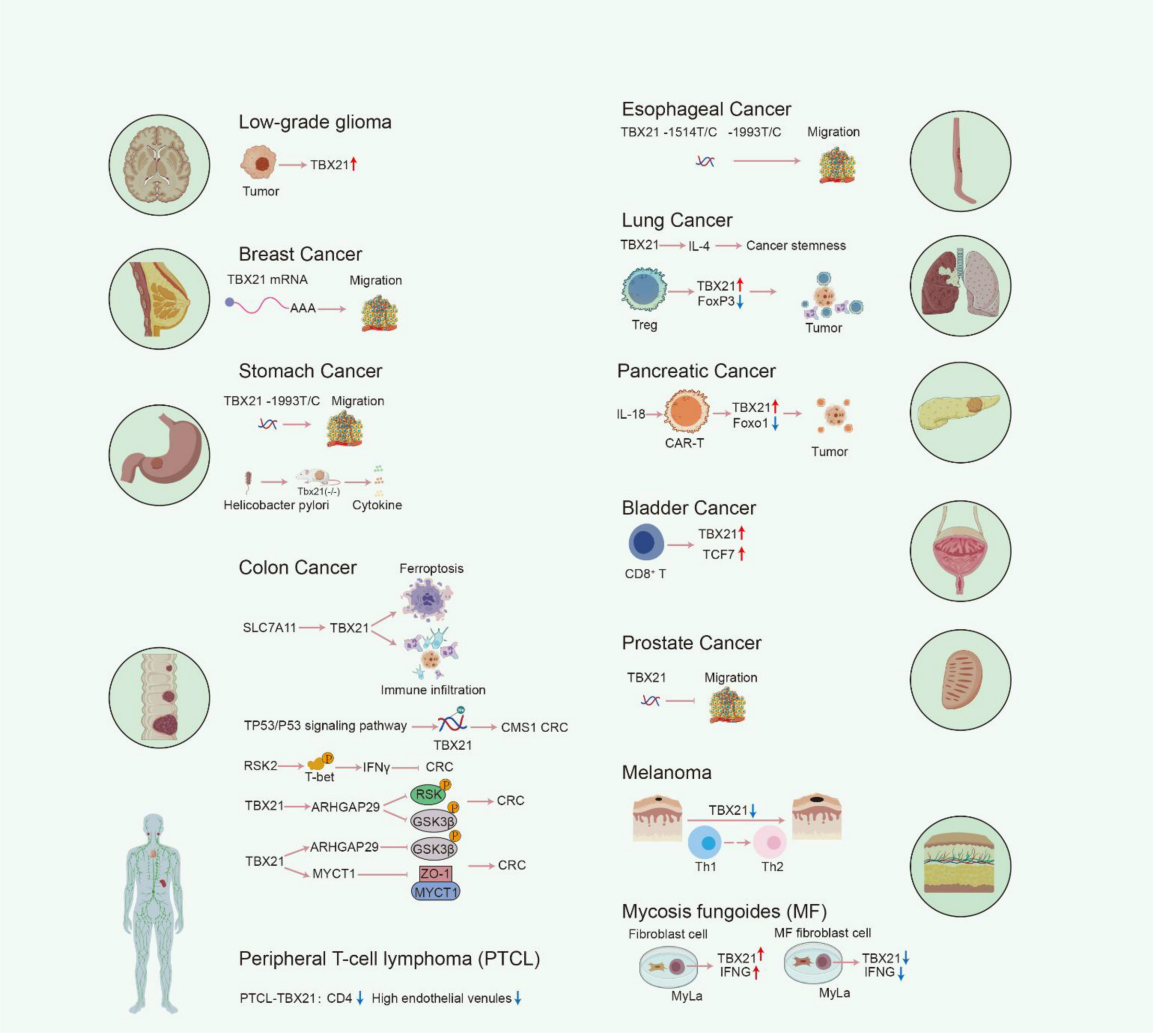
The function and molecular mechanisms of T-bet in tumor pathologies.

Genetic polymorphisms in *TBX21* are associated with increased gastric cancer risk. The encoded T-bet protein participates in disease progression and distant metastasis by regulating Th1 immune responses and cytokine secretion. A population-based case-control study conducted by Le-Hui Zhang et al. revealed a significant increase in the risk of gastric cancer associated with the -1993CC genotype when compared to the *TBX21* -1993TT genotype. This finding indicates that host *TBX21* -1993T/C polymorphisms are linked to a heightened risk of gastric cancer, particularly in cases involving distant metastasis (111). In the context of antitumor immunity, the local progression or distant metastasis of primary tumors is modulated by type 1 immune responses, predominantly through the IFN-γ signaling pathway (112) (113) (114). The secretion of IFN-γ is largely reliant on helper T cells, and helper T cell-mediated type 1 IFN-γ-associated responses enhance tumor immunogenicity while inhibiting tumor cell progression. In *Tbx21* knockout mice, the expression levels of cytokines such as IFN-γ, TNF-α, IL-1β, IL-12, and IL-13 were markedly different from those observed in wild-type mice (115) (116), with a significant reduction in these cytokines noted in the *Tbx21* knockout models. Given that Helicobacter pylori infection is a prominent risk factor for gastric cancer, T-bet appears to play a crucial role in H. pylori-induced gastric cancer in murine models (117). This section provides a comprehensive overview of the involvement of T-bet in the pathogenesis of various diseases across multiple biological systems, including autoimmune disorders, infectious diseases, allergic conditions, endocrine disorders, psychiatric illnesses, and chromosomal abnormalities. The critical function of T-bet in the development of non-neoplastic diseases and the underlying pathogenic mechanisms are discussed in detail (**Figure 5**).

### 5.2 Colorectal cancer

T-bet serves as a key tumor suppressor in colorectal cancer. Its downregulation correlates with disease progression and inhibits tumor growth and metastasis by regulating immune responses and signaling pathways such as RSK2/T-bet/IFN-γ and ARHGAP29/GSK3β. In their research, Xin Cheng et al. demonstrated that SLC7A11 is overexpressed in colon adenocarcinoma, with its expression levels exhibiting a significant correlation with the infiltration of CD8^+^ T cells, neutrophils, and DCs within colon cancer tissues. The study further revealed a strong association between the Th1 cell infiltrating lymphocyte marker, T-bet, and SLC7A11 expression. Additionally, five hub genes co-expressed with SLC7A11 that are implicated in the induction of ferroptosis were identified. Consequently, T-bet may play a mediating role in the function of SLC7A11 concerning ferroptosis induction and the regulation of tumor immunity (118). Similarly, Yuanyuan Shen et al. reported that patients with CMS type 1 colon cancer exhibiting high T-bet expression and low methylation levels experienced a significant survival advantage. Furthermore, T-bet was found to correlate with markers indicative of enhanced cytotoxic T lymphocyte (CTL) functionality. The study also suggested that the TP53/P53 signaling pathway might contribute to the deceleration of colon cancer progression by altering *TBX21* methylation, thereby upregulating *TBX21* expression in CTL cell (119). Moreover, Ke Yao et al. observed a reduction in IFN-γ secretion by peripheral blood mononuclear cells at various stages of colon cancer metastasis when compared to healthy controls. *In vitro* kinase assays indicated that RSK2 phosphorylated T-bet at series 498 and 502, which subsequently activated the transcription of the IFN-γ gene. In summary, these findings imply that the phosphorylation of T-bet is essential for the inhibition of colon cancer metastasis and growth, facilitated by the positive regulation of the RSK2/T-bet/IFN-γ signaling pathway in CD4^+^ T cell and CD8^+^ T cell (120) (**Figure 5**).

As research into tumor immunity continues to advance, the role of anti-tumor immune responses in tumor progression has become increasingly significant. Among the immune-related molecules, T-bet has garnered considerable attention in the context of tumors. A study conducted by Xinyu Jiang et al. revealed that T-bet expression in colorectal cancer (CRC) tissues was markedly lower than in normal tissues and exhibited a negative correlation with the TNM staging of CRC. The ectopic expression of T-bet was found to inhibit cell proliferation and promote apoptosis. Furthermore, xenograft models demonstrated that T-bet impedes the progression of colorectal cancer through the ARHGAP29/RSK/GSK3β signaling pathway *in vivo* (121). And, in our investigation, we corroborated that T-bet expression in colorectal cancer tissues was significantly diminished compared to normal tissues and was inversely related to TNM stage. Ectopic expression of T-bet was shown to inhibit the migration of colorectal cancer cells *in vitro* and metastasis *in vivo*. Additionally, analysis using a human phosphokinase array indicated that T-bet expression led to a reduction in the phosphorylation of GSK3β. RNA sequencing identified *ARHGAP29* and *MYCT1* as potential target genes associated with T-bet. Notably, T-bet was found to directly bind to the promoter of *ARHGAP29*, thereby upregulating its expression, which subsequently upregulates T-bet itself. This interaction inhibits GSK3β phosphorylation. Concurrently, T-bet enhances MYCT1 expression, facilitating its interaction with ZO-1 to regulate the cytoskeleton. The pathways involving ARHGAP29/GSK3β and MYCT1/ZO-1 collaboratively function to suppress the metastasis of colorectal cancer cells. Conversely, the knockdown of T-bet, ARHGAP29, or MYCT1, or the application of LiCl (which enhances GSK3β phosphorylation), negated the T-bet -mediated inhibition of colorectal cancer cell migration both *in vitro* and *in vivo*. These findings indicate that T-bet plays a crucial role in inhibiting the proliferation and metastasis of colorectal cancer while promoting apoptosis (122), thereby positioning T-bet as a potential therapeutic target for colorectal cancer treatment (**Figure 5**).

### 5.3 Lung cancer and pancreatic cancer

T-bet exhibits dual roles in lung cancer: it promotes lung adenocarcinoma progression by maintaining tumor stemness and activating the T-bet-IL-4 signaling pathway, while simultaneously being highly expressed in TAA-specific tumor-infiltrating Treg (TIL-Treg) within the tumor microenvironment, potentially contributing to antitumor immune responses. Shuangtao Zhao et al. demonstrated that the upregulation of *TBX21* is correlated with unfavorable prognostic outcomes in patients with lung adenocarcinoma, thereby establishing *TBX21* upregulation as a negative prognostic biomarker for this patient population. Furthermore, the downregulation of T-bet in lung cancer cells resulted in a reduction in the proportion of cancer stem cells, as well as a decrease in their sphere formation and tumor initiation frequencies, indicating that T-bet plays a pivotal role in sustaining cancer stemness. Additionally, it has been observed that the activation of T-bet initiates the TBX21-IL-4 signaling pathway, which facilitates tumor initiation, promotes tumor growth, and enhances the expression of stemness markers (110). In a separate study conducted by Arbor G. Dykema et al., an analysis of TIL-Treg from both treated and untreated non-small cell lung cancer patients receiving anti-PD-1 therapy revealed subpopulations of TIL-Treg that exhibited elevated levels of TNFRSF4 (OX40) and TNFRSF18 (GITR). These subpopulations demonstrated the most significant inhibitory capabilities *in vitro* and were associated with resistance to PD-1 blockade. In a murine tumor model, it was found that nearly all TIL-Treg expressing specific T cell receptors were directed against tumor-associated antigens. Within two weeks of tumor infiltration, these TIL-Treg developed a distinct Th1-like phenotype characterized by the downregulation of FoxP3 and the upregulation of T-bet, IFN-g, and various pro-inflammatory granzymes. The Treg subpopulations observed in mouse tumor models exhibited a high degree of similarity to those found in human tumors responsive to anti-PD-1 therapy (123). These findings suggest that elevated expression of T-bet in TAA-specific TIL-Treg may enhance anti-tumor immune responses. Moreover, a systematic screening conducted by Markus Chmielewski et al. indicated that IL-18 can polarize chimeric antigen receptor (CAR) T cells towards a phenotype characterized by high T-bet and low FoxO1, which is associated with acute inflammatory responses and demonstrates efficacy in the treatment of large pancreatic and lung tumors (124) (**Figure 5**).

### 5.4 Prostate cancer and bladder cancer

In a transgenic mouse model of prostate cancer expressing T-bet, it has been observed that T-bet influences the incidence of primary tumors and contributes to a moderate decrease in tumor progression rates (125). Furthermore, T-bet demonstrates a pronounced inhibitory effect primarily on cancer metastasis, in addition to its moderate impact on primary tumors. *TBX21* in CD8^+^ T cells is recognized as a key regulator of tumor immune responses, suppressing prostate cancer metastasis by promoting immune surveillance. These results provide compelling evidence that the regulation of T-bet within the immune response is linked to cancer risk and may elucidate certain aspects of the risk associated with cancer metastasis (31). In a related study, Jie Wang et al. assessed the transcription factor activities of various immune cell types in bladder cancer and identified significant enrichment of TCF7 and T-bet in CD8^+^ T cells (126) (**Figure 5**).

### 5.5 Breast cancer

*TBX21* is significantly expressed in breast cancer and serves as a key biomarker for predicting disease recurrence. Through the examination of mRNA expression profiles from 15 normal samples and 669 breast cancer patients, three mRNAs—*TBX21*, *TGIF2*, and *CYCS*—were found to be significantly expressed. Subsequently, a model incorporating these three mRNAs was established (127). This model serves as a dependable instrument for the precise prediction of disease recurrence and enhances the predictive accuracy regarding survival probabilities in patients, regardless of the presence of metastatic breast cancer (**Figure 5**).

### 5.6 Esophageal cancer

Polymorphisms in the *TBX21* gene may increase susceptibility and progression risk for esophageal squamous cell carcinoma by affecting immune cell function. Huihui Li et al. identified a correlation between the *TBX21* -1514T/C and -1993T/C polymorphisms and the occurrence of lymph node or distant metastasis in esophageal squamous cell carcinoma (ESCC). Specifically, the ACC, ACT, and ATC haplotypes derived from the *TBX21* gene were associated with an increased susceptibility to ESCC within a high-risk population in China (128). Conversely, a study conducted by Cihan Uslu et al., which involved 35 patients diagnosed with laryngeal cancer and 35 control volunteers, found no significant impact of the *TBX21* rs17250932 polymorphism on the development of laryngeal cancer (129) (**Figure 5**).

### 5.7 Low-grade glioma

*TBX21* plays a crucial role in the immune regulation of LGG. Risk models developed to incorporate *TBX21* as an inflammation-associated gene in LGG demonstrated significant correlations with various parameters, including levels of immune cell infiltration, tumor mutational burden, expression of HLA and immune checkpoint genes, tumorigenicity scores, and indices of tumor stemness in LGGs (130). Notably, *ADRB2, CD274, IL12B, PRF1, TNFRSF11B*, and *TTR* exhibited high expression levels in immune cells, while *TBX21* was predominantly expressed in malignant cells. Additionally, SFTPC showed elevated expression in oligodendrocytes, CXCL12 was highly expressed in both malignant and immune cells, and NFE2L2 was significantly expressed in both oligodendrocytes and immune cells (**Figure 5**).

### 5.8 Skin cutaneous melanoma

*TBX21* expression undergoes dynamic changes in melanoma, with low levels indicating poor prognosis. It participates in disease progression by regulating immune balance and cellular malignant behavior. Zhang et al. demonstrated that *TBX21* expression is markedly elevated in SKCM tissues, with a progressive decline observed as the disease advances. Kaplan-Meier survival analysis indicated that SKCM patients exhibiting low levels of *TBX21* expression experience poorer prognoses compared to those with elevated *TBX21* levels (131). T-bet, a transcription factor, is instrumental in regulating the balance between Th1 and Th2 cell populations (132), and it has been noted that tumor progression, particularly in melanoma, is frequently linked to a shift from Th1 to Th2 dominance (133) (134). Additionally, T-bet proteins are critical in mediating the metastatic disease process in melanoma through NK cells. Through the collection and processing of single-cell source data, Zhao et al. further categorized a total of 10,137 melanoma cells into seven distinct subtypes: C0 melanoma BIRC7, C1 melanoma CDH19, C2 melanoma EDNRB, C3 melanoma BIRC5, C4 melanoma CORO1A, C5 melanoma MAGEA4, and C6 melanoma GJB2. Notably, the C4 melanoma subtype, characterized by CORO1A, may exhibit heightened sensitivity to NK and T cell-mediated immune responses, while other subtypes may demonstrate increased resistance to NK cell activity. Transcription factor enrichment analysis identified *TBX21* as the predominant transcription factor in the CORO1A subtype of C4 melanoma, which is also associated with the M1 immune module. Furthermore, *in vitro* studies revealed that the knockdown of *TBX21* significantly impaired melanoma cell proliferation, invasion, and migration (135) (**Figure 5**).

### 5.9 Other neoplastic diseases

Within the framework of the tumor microenvironment, Treg cells have been identified in various malignancies, including ovarian, lung, colorectal, hepatocellular, and oral squamous cell carcinomas (136) (137) (123). These Tregs are distinguished by the expression of T-bet, increased levels of Helios and CXCR3, and exhibit significant inhibitory activity in ex vivo assays. In a murine fibrosarcoma model induced by 3-methylanthracene, Th1-like Tregs (characterized as T-bet^+^ CXCR3^+^) were found to be enriched and demonstrated a high rate of proliferation within the tumor infiltrate (138).

### 5.10 Hematologic neoplasms

Peripheral T-cell lymphomas (PTCL) represent a heterogeneous collection of clinicopathological entities that are typically associated with an aggressive clinical trajectory. Among these, angioimmunoblastic T-cell lymphoma (AITL) and PTCL not otherwise specified (PTCL-NOS) are the two predominant categories. Recent advancements in gene expression profiling (GEP) have led to the identification of novel PTCL subgroups, specifically PTCL-*GATA3* and PTCL-*TBX21*. Research conducted by Tayla B. Heavican et al. revealed that PTCL-GATA3 is characterized by a more intricate genomic landscape, marked by frequent deletions or mutations in oncogenes that affect the CDKN2A/B-TP53 axis and the PTEN-PI3K pathway. Conversely, the *TBX21* subgroup exhibits fewer gene copy number alterations, predominantly affecting cytotoxic effector genes, and is enriched in mutations within genes that govern DNA methylation. Furthermore, Bogung Han et al. reported that *TBX21* isoforms were more prevalent than *GATA3* isoforms in AITL, with *GATA3* isoforms correlating with a poorer overall survival compared to *TBX21* isoforms (139). In summary, PTCL subgroups can be classified based on the presence or absence of *TBX21*, suggesting that these distinct subgroups may have evolved through divergent genetic pathways, thereby providing a biological basis for potential therapeutic interventions to be explored in future clinical trials. Additionally, a study by Yasumasa Shimasaki et al. indicated that PTCL-T-bet exhibited significantly lower CD4 T cell positivity, reduced counts of high endothelial venules, and a more favorable response to initial therapy. Gene expression analysis utilizing the nCounter system demonstrated that the expression levels of tumor immunity-related genes (such as *PD-L1, LAG3*, and *IDO1*) were elevated in PTCL-*TBX21* compared to PTCL-*GATA3*. Notably, overall survival was significantly diminished in the PTCL-*GATA3* cohort relative to the PTCL-*TBX21* group (140). Consequently, the classification of PTCL-*NOS* into PTCL-*TBX21* and PTCL-*GATA3* may enhance prognostic predictions for Japanese patients and facilitate the stratification of tumor immune checkpoint inhibitor therapies in clinical settings (**Figure 5**).

Mycosis fungoides (MF) is classified as a primary cutaneous T-cell lymphoma (CTCL). A recent investigation into the interactions between fibroblasts and malignant T-cells, specifically MyLa cells, within the tumor microenvironment revealed that MyLa cells co-cultured with normal fibroblasts exhibited an upregulation of IFNG and T-bet expression. Conversely, when MyLa cells were co-cultured with MF fibroblasts, there was a notable inhibition of IFNG and T-bet expression (141). These co-culture models demonstrate that normal and MF fibroblasts exert differential effects on T cell phenotypes, particularly in the regulation of Th1 cytokine expression, thereby highlighting their distinct roles in the progression of MF (**Figure 5**).

## 6 T-bet and tumor-associated signaling pathways

### 6.1 Regulation of immune cells and cellular cytokines by T-bet

**Figure 6:**
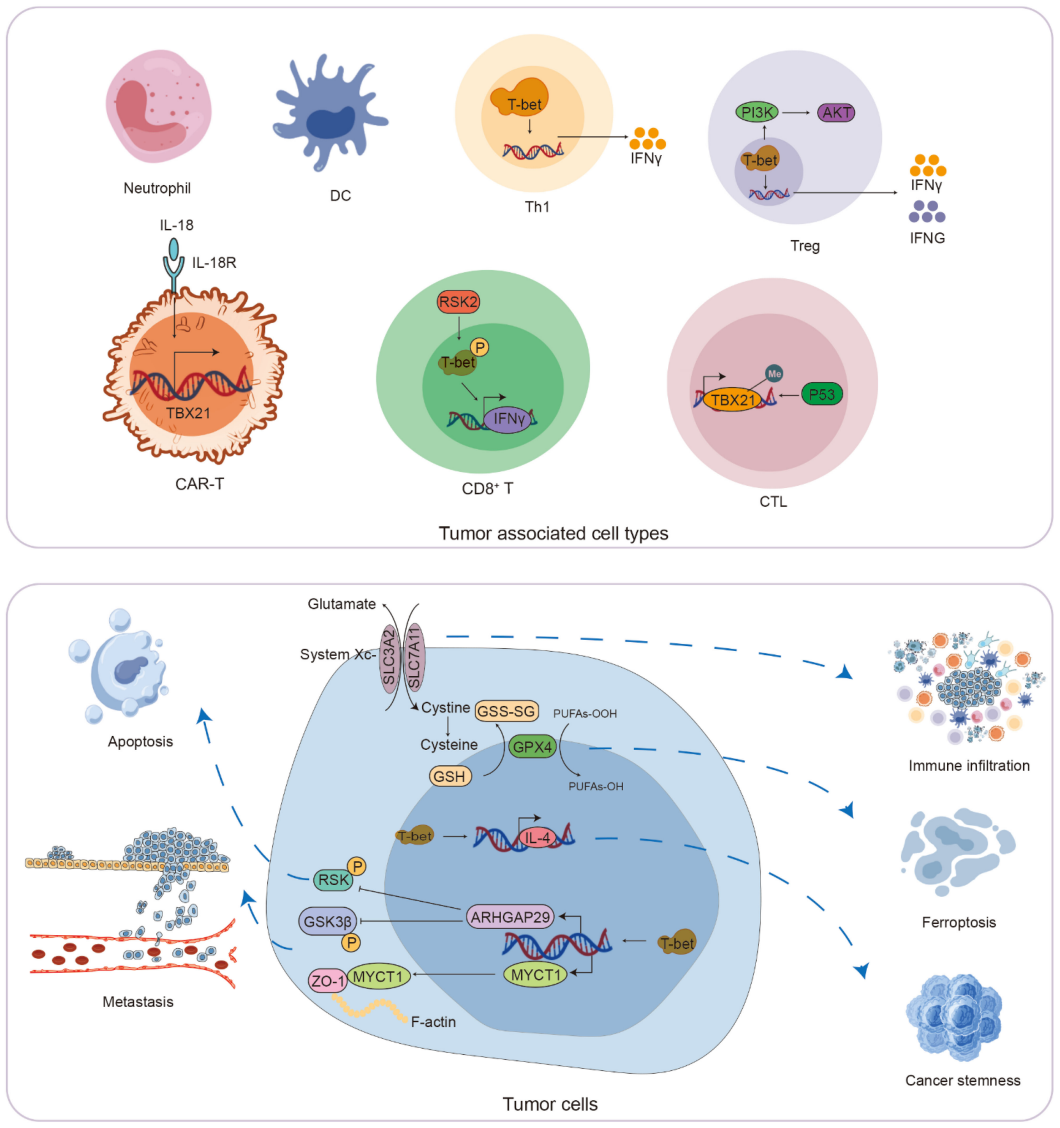
The role of T-bet-associated immune cells and signaling pathways in the process of tumorigenesis.

In the context of tumors, T-bet is implicated in the differentiation of various immune cell types and exhibits a significant correlation with the infiltration levels of immune cells, including CD8^+^ T cells, neutrophils, DCs, B cells, and NK cells. T-bet facilitates the differentiation of Th1 cells, modulates the expression of cytokines such as IFN-γ, and activates immune cells to target and eliminate tumor cells. For instance, in gastric cancer, T-bet is instrumental in mediating IFN-γ production, thereby playing a critical role in anti-tumor immunity. In melanoma, IFN-γ enhances the capacity of immune cells to recognize and destroy tumor cells. CAR T cells that exhibit high levels of T-bet have demonstrated efficacy in the treatment of lung and pancreatic cancers. Furthermore, T-bet influences the expression of various cytokines within tumor cells. In lung cancer, the activation of T-bet initiates the TBX21-IL-4 signaling pathway, which promotes the expression of markers associated with tumor initiation, growth, and stemness. In the case of mycosis fungoides, MyLa cells co-cultured with normal fibroblasts showed an increase in the expression of IFNG and T-bet, whereas co-culture with mycosis fungoides fibroblasts resulted in the suppression of both IFN-g and T-bet expression (**Figure 6**).

### 6.2 T-bet and PI3K/AKT signaling pathway

In the context of highly immunogenic melanoma, the blockade of PD-1 facilitates the proliferation of a particularly active subset of Treg that co-express PD-1 and Helios. While these Treg cells exhibit strong suppressive capabilities in non-tumor environments, the tumor microenvironment, characterized by various stimuli and inflammatory signals, induces a local transformation of these Treg cells, endowing them with Th1-like characteristics. This transformation is marked by the expression of T-bet and gamma interferon, an increase in PI3K/AKT signaling activity, and a subsequent loss of their suppressive function over effector T cells (142) (**Figure 6**).

### 6.3 T-bet and Ferroptosis-related signaling pathway

Ferroptosis represents a regulated mechanism of cellular demise, distinguished by the iron-dependent accumulation of lipid peroxidation to cytotoxic levels. The System Xc- transporter, composed of SLC3A2 and SLC7A11 dimers, is situated within the cell membrane. SLC7A11 serves as the primary subunit responsible for the translocation of cystine into the cytosol, facilitating the synthesis of glutathione (GSH). Consequently, the downregulation of SLC7A11 expression can trigger ferroptosis. The induction of ferroptosis in neoplastic cells presents a promising strategy for cancer therapy. Certain pharmacological agents can induce ferroptosis in tumor cells by either depleting intracellular glutathione or directly inhibiting GPX4 activity. In the context of colon cancer, a significant correlation has been observed between T-bet expression and SLC7A11 levels, as well as with the infiltration of medium CD8 T cells, neutrophils, and dendritic cells. Therefore, SLC7A11 emerges as a potential therapeutic target for colon adenocarcinoma through the induction of ferroptosis (**Figure 6**).

### 6.4 T-bet and methylation-related signaling pathway

Methylation represents a prevalent form of epigenetic modification characterized by the addition of methyl groups to specific regions of DNA, typically within CpG islands, and is predominantly facilitated by DNA methyltransferases. This modification is intricately linked to processes such as tumorigenesis and cancer progression. In healthy cells, the maintenance of appropriate methylation patterns is crucial for the preservation of normal cellular functions. Deviations from these methylation patterns can lead to gene abnormalities or even genomic instability, resulting in uncontrolled cellular behavior, which is significantly associated with the development of tumors. For instance, the TP53/P53 signaling pathway may enhance the expression of T-bet, thereby contributing to the attenuation of colon cancer progression through the modulation of *TBX21* methylation (**Figure 6**).

### 6.5 T-bet and RSK/GSK3β-related signaling pathway

In the context of colon cancer, T-bet has been shown to inhibit the proliferation of colon cancer cells and promote apoptosis via the ARHGAP29/RSK/GSK3β signaling pathway *in vivo*, thereby impeding the progression of colorectal cancer. Furthermore, T-bet directly interacts with the promoter of ARHGAP29, leading to its upregulation, which subsequently inhibits the phosphorylation of GSK3β. Concurrently, T-bet enhances the expression of MYCT1, facilitating its interaction with ZO-1 to modulate the cytoskeletal structure. The combined effects of the ARHGAP29/GSK3β and MYCT1/ZO-1 pathways serve to inhibit the metastasis of colorectal cancer cells. Additionally, RSK2 phosphorylates T-bet at serine residues 498 and 502, which activates the transcription of the *IFN-γ* gene, thereby further inhibiting the metastasis and growth of colon cancer (**Figure 6**).

## 7 Immunotherapy and future prospect

T-bet is extensively acknowledged as a critical effector protein implicated in autoimmune diseases, allergic conditions, and malignancies. Its expression within the immune system is essential for mediating anti-infective responses and facilitating tumor immunity. Nonetheless, the intricate nature and inherent limitations of its clinical application warrant comprehensive evaluation.

**Figure 7:**
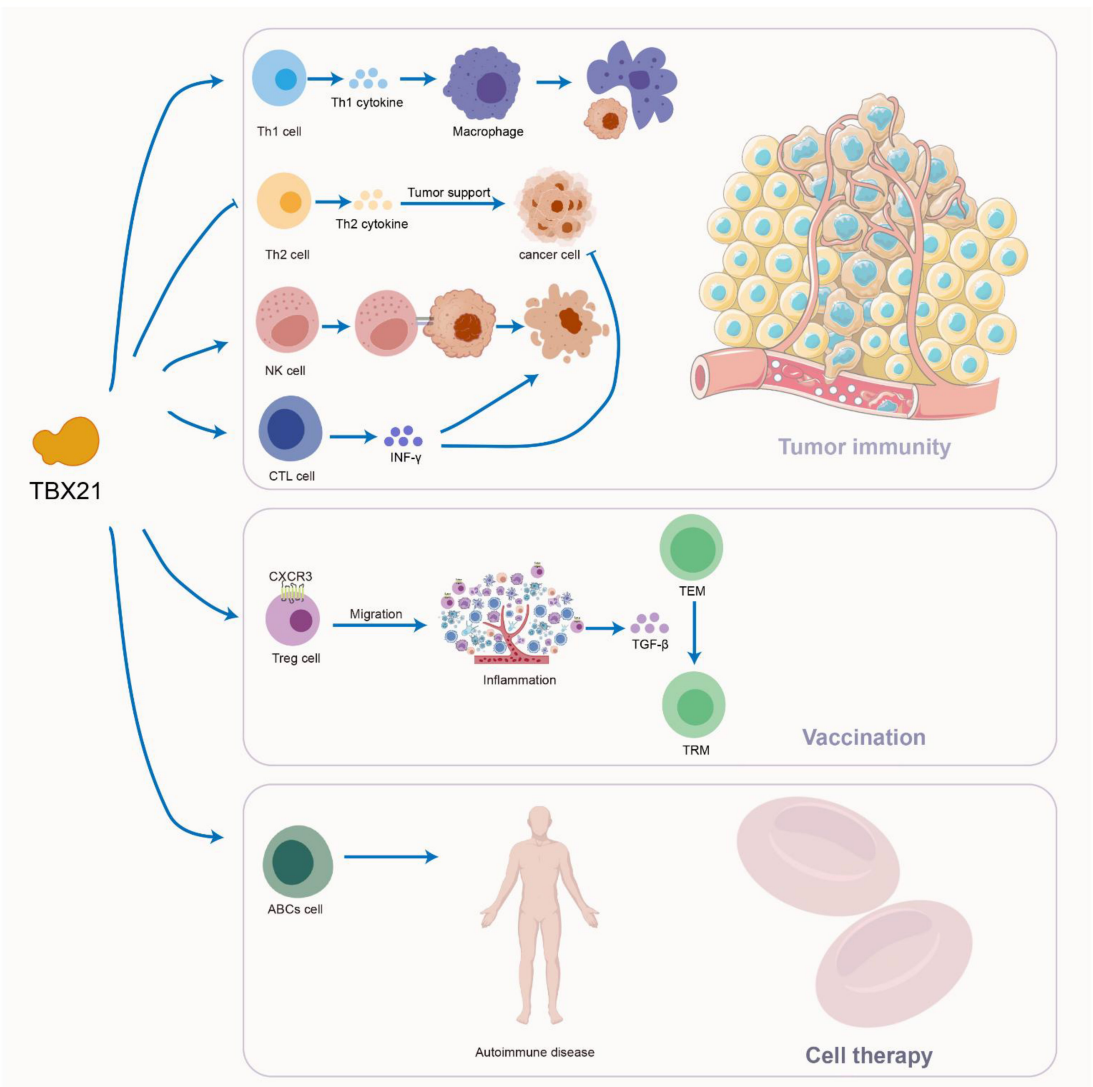
Immunotherapeutic approaches associated with T-bet.

T-bet plays a fundamental role in preserving the homeostatic balance between the host and microbiota in the colon, serving as a principal regulator of the intestinal microenvironment. Notably, its expression demonstrates a U-shaped association with intestinal inflammation: overexpression compromises epithelial barrier integrity and disrupts ion channel function, whereas insufficient expression results in dysregulated Th1/Th17 immune responses and heightened susceptibility to infections (132). The status of T-bet is also crucial for cancer progression and prognosis, with research indicating its involvement in modulating the balance of helper T cell subsets. T-bet promotes the differentiation of Th1 cells, which are responsible for producing cytokines that activate immune cells, such as macrophages, thereby enhancing their capacity to phagocytose tumor cells. Additionally, T-bet inhibits the differentiation of Th2 cells by downregulating GATA3 expression and reducing GATA3’s binding affinity to DNA (143) (144). GATA3 is a key factor in the differentiation of naïve CD4^+^ T cells into Th2 cells, and a Th2-dominant cytokine profile is known to support tumor growth (145) (146). Dysregulation of the Th2 response may lead to diminished antibody production and a compromised immune response against viral infections. However, clinical interventions targeting this pathway must be approached with caution, as excessive suppression of Th2 cells may compromise host defense against helminth infections and disrupt the secretion balance of Th2 cytokines (e.g., IL-4, IL-13) in patients with allergic diseases such as asthma, potentially facilitating immune evasion within the tumor microenvironment. Moreover, T-bet facilitates the differentiation and functionality of CTL and NK cells. In the context of tumor therapy, enhancing the activity of these cells is beneficial for the recognition and elimination of tumor cells. For instance, T-bet drives CTL cells to secrete IFN-γ, a cytokine that inhibits tumor cell proliferation, promotes apoptosis, and augments immune surveillance of tumors. By modulating the expression or activity of T-bet, it is anticipated that the anti-tumor immune response can be rebalanced, thereby improving the immune system’s capacity to eradicate tumors (**Figure 7**). Nevertheless, therapeutic approaches targeting T-bet remain in the experimental stage, with no specific modulators currently available. While small-molecule inhibitors such as JAK inhibitors may indirectly influence the T-bet signaling pathway, their off-target effects—including inhibition of Th17 differentiation—pose risks of opportunistic infections. Additionally, the relationship between T-bet expression levels and patient prognosis exhibits considerable heterogeneity across various tumor types, underscoring the necessity for multi-omics analyses to identify patient subgroups most likely to benefit from T-bet-targeted interventions.

Recent research has identified T-bet^+^ Treg cells as a crucial cell population in the generation and establishment of CD8^+^ tissue-resident memory (TRM) cells. TEM cells undergo differentiation into TRM cells in a manner dependent on TGF-β. In the context of inflammation, T-bet^+^ CXCR3^+^ Tregs migrate to inflamed peripheral tissues, where they colocalize with CXCR3^+^ CD8^+^ TEM cells. Within these inflamed tissues, T-bet^+^ Tregs produce and process TGF-β, which promotes the expression of CD103 on TEM cells and facilitates their further differentiation into TRM cells. The establishment and differentiation of TRM cells are critical for local immunoprotection, which has become a focal point for the development of novel vaccines (147). A promising strategy to enhance this process involves a dual approach that targets local immune memory while simultaneously inducing T-bet^+^ Tregs. Nonetheless, translating these strategies to clinical application in humans presents several challenges, including limited control over the tissue microenvironment and inter-individual variability. Current studies predominantly utilize specific experimental models, whereas the heterogeneity inherent to human tissues may result in tissue-specific differences in TGF-β-dependent differentiation pathways. Consequently, investigating the molecules involved in Treg development, stability, and inhibition, alongside advancements in next-generation biotechnology, will facilitate the intelligent design of effective and more targeted immunotherapies that leverage the use and modification of Tregs (**Figure 7**).

Previous research has established that the antitumor efficacy of NK cell-based immunotherapy is diminished, a phenomenon closely linked to the downregulation of the transcription factor T-bet. Consequently, it is imperative to elucidate the mechanisms responsible for T-bet suppression in tumor-associated NK cells and to devise approaches to restore its expression, thereby maintaining the antitumor functionality of NK cells (148). In this context, certain small molecules have been identified that may enhance T-bet expression. Additionally, the ectopic expression of specific transcription factors in immune cells utilized for tumor immunotherapy has been documented (149, 150). Notably, CAR-T cells that overexpress T-bet exhibit a Th1 phenotype, which is characterized by heightened anti-tumor activity and improved survival rates in tumor-bearing murine models. Nevertheless, the safety profile and long-term efficacy of these modified cells in human subjects remain to be validated through extensive clinical trials. These findings imply that the ectopic expression of T-bet in NK cells may hold promise for future tumor immunotherapy approaches. Furthermore, leveraging our understanding of the cytokine receptors and signaling pathways that facilitate T-bet induction, we can strategically design the signaling domains of CAR-NK cells to activate the T-bet-inducing pathway, thereby enhancing NK cell effector functions (151). Alternatively, the development of multi-specific antibodies that activate T-bet-inducible cytokine receptors could also be employed to promote T-bet expression (**Figure 7**). For clinical translation, these strategies must overcome several challenges, including the precise modulation of signaling domains to avoid cytotoxicity resulting from excessive activation, as well as ensuring the stability and durability of genetic modifications. Similarly, the production of multi-specific antibodies targeting T-bet-induced receptors entails practical difficulties such as manufacturing complexity, potential immunogenicity, and considerations of cost-effectiveness.

In B cells, T-bet plays a crucial role in isotype switching to specific IgG subclasses, namely IgG2a/c in murine models and IgG1/3 in humans. In the context of various autoimmune diseases, including systemic lupus erythematosus and rheumatoid arthritis, there is an expansion of specific subpopulations of T-bet-expressing B cells, referred to as age-associated B cells (ABCs) and double-negative B cells (DNs). These cell populations have been implicated in the pathogenesis of autoimmune disorders in both murine and human subjects (152). Given the pathogenic significance of ABCs and DNs in systemic autoimmune diseases, numerous research groups have directed their efforts toward developing therapeutic strategies that specifically target these B cell populations. While various approaches aimed at T-bet^+^ B cells have demonstrated efficacy in reducing the prevalence of ABCs and DNs, they also have broader implications for the overall immune system. Consequently, it is imperative to introduce novel targeted therapies into clinical practice to optimize therapeutic outcomes for patients with autoimmune conditions (**Figure 7**).

*TBX21* demonstrates considerable potential within the field of tumor immunotherapy. An in-depth investigation into the molecular mechanisms by which *TBX21* regulates immune cell functions in the tumor microenvironment is expected to provide valuable insights for the discovery of novel immunotherapeutic targets. Specifically, targeted modulation of the *TBX21* signaling pathway may potentiate the antitumor activity of cytotoxic T lymphocytes and natural killer cells, thereby enhancing their ability to selectively eradicate tumor cells and addressing the common challenge of drug resistance in immunotherapy. Moreover, the integration of gene editing technologies with cell-based therapeutic approaches to precisely alter the *TBX21* gene in immune cells could substantially improve their antitumor efficacy. This strategy offers the potential to develop engineered immune cells with superior tumor-targeting and clearance capabilities, representing a promising new avenue in tumor immunotherapy. Additionally, exploiting the immunomodulatory properties of *TBX21* may facilitate the creation of immunoadjuvants or combination therapies that enhance the effectiveness of existing immunotherapeutic modalities, broaden their clinical applicability, and provide renewed therapeutic options for a wider range of cancer patients. Collectively, these advances have the potential to significantly propel the field of tumor immunotherapy forward.

## 8 Conclusions

**Figure 8:**
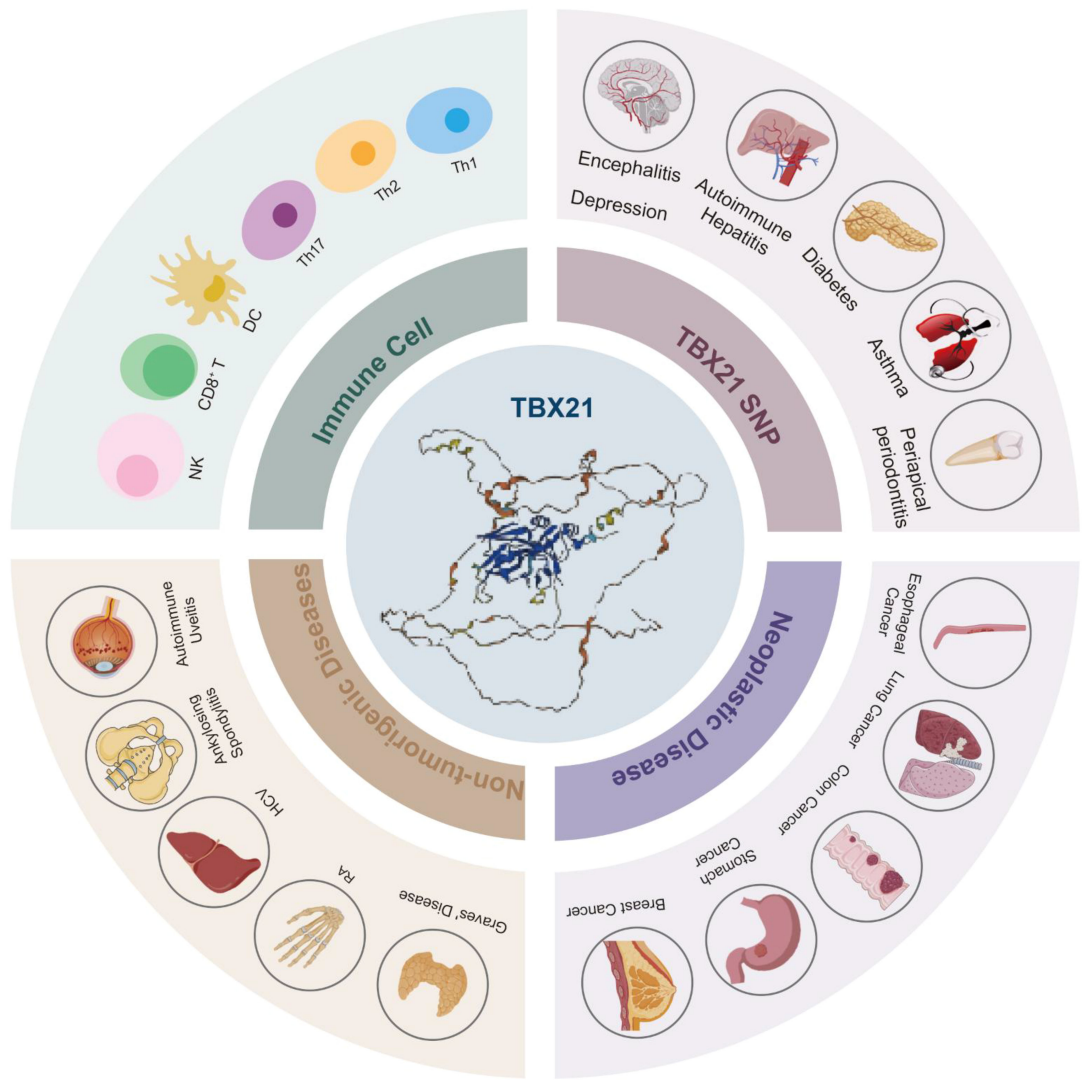
Proposed model of T-bet functions, mechanisms and applications.

In summary, this review integrates recent research findings to systematically delineate the role of *TBX21* in the pathogenesis of a broad spectrum of systemic diseases, including autoimmune disorders, infectious diseases, allergic conditions, endocrine dysfunctions, psychiatric disorders, and chromosomal abnormalities. Additionally, it highlights *TBX21*’s involvement in the progression of various malignancies, such as esophageal, gastric, breast, colorectal, and prostate cancers, as well as hematological neoplasms. The review further examines the principal molecular mechanisms by which *TBX21* contributes to these disease processes and proposes potential pathways for targeted therapeutic interventions (**Figure 8**). Moreover, the analysis extends to the function of *TBX21* in tumor biology, encompassing tumor cell proliferation, apoptosis, EMT, metastasis, immune cell infiltration, and ferroptosis, evaluating its utility as both a prognostic biomarker and a therapeutic target (**Figure 8**).

Nonetheless, it is imperative to acknowledge several significant limitations associated with the current clinical assertions. Primarily, the majority of existing studies are based on *in vitro* experiments or animal models, with a paucity of validation in large-scale clinical cohorts, thereby leaving the precise mechanisms and clinical relevance of *TBX21* in human diseases insufficiently characterized. Furthermore, therapeutic approaches targeting *TBX21* remain in nascent stages of development and face considerable challenges, including suboptimal drug delivery efficiency, potential off-target effects, and the inherent complexity of the tumor microenvironment, all of which impede clinical translation. In addition, inconsistencies in the reported associations between *TBX21* expression and disease prognosis across studies may be attributable to sample heterogeneity, methodological discrepancies, and confounding variables, underscoring the need for further standardization and rigorous validation. Finally, immune-related adverse effects and long-term safety profiles have not been comprehensively assessed, potentially limiting the broader clinical application of *TBX21*-targeted therapies in immune-mediated diseases and cancer.

Despite these constraints, this review makes a substantive contribution to the extant literature by enhancing the understanding of *TBX21*’s multifaceted role in disease progression, particularly within the contexts of oncology and immune regulation. From a clinical perspective, the findings suggest that *TBX21* holds considerable promise as a biomarker for the diagnosis and treatment of immune-related diseases and malignancies, contingent upon the resolution of the aforementioned challenges. Moreover, this work provides a foundation for the development of novel immunotherapeutic strategies targeting *TBX21*.
